# Exploring secular variation of the gravitational constant from high-resolution quasar spectra

**DOI:** 10.1038/s41598-024-65484-5

**Published:** 2024-07-06

**Authors:** T. D. Le

**Affiliations:** 1 Division of Applied Physics, Dong Nai Technology University, Bien Hoa City, Vietnam; 2 Faculty of Engineering, Dong Nai Technology University, Bien Hoa City, Vietnam

**Keywords:** Quasars, Varying-gravitational constant, Quasar HE 0515–4414, Absorption spectra analysis, GUTs, General relativity and gravity, Time-domain astronomy

## Abstract

The exploration of potential variations in fundamental physical constants is crucial for testing of Grand Unification Theories (GUTs), which aim to unify the fundamental forces of nature. This study utilizes direct observational tests to explore these variations, offering a deep-look into the universe's distant past. By analyzing high-resolution quasar spectra of HE 0515–4414^*^ and comparing them with laboratory-calibrated Ritz wavelengths, we establish an upper limit on the possible cosmological deviation of the gravitational constant: $\dot{\text{G}}/\text{G}=(0.918 \pm 2.830)\times {10}^{-15}{\text{ yr}}^{-1}$ over cosmic timescales. Our findings provide a novel tool for probing the physical implications of GUTs, contributing to our understanding of fundamental physics.

## Introduction

Unification scenarios are fundamental tests of our current understanding of physics. One significant aspect of this pursuit is investigating potential variations in fundamental physical constants, particularly the gravitational constant $G$^1–5^. These constants play a crucial role in the overarching goal of unifying the four fundamental forces in nature^6,7^. Modern physics theories, such as M-theory, string theory, and superstring theory^8^, offer frameworks where variations in these constants might naturally occur^9,10^. In these theories, there is a postulated mechanism where the fine-structure constant α, the proton-to-electron mass ratio µ, and the gravitational constant $G$ may vary differently with the square of the mean scale of extra dimensions. The evolution of these dimension scales is intricately connected to variations in space and time^11–14^. Notably, recent observations have confirmed variations in α over cosmological timescales, indicating that its value has evolved throughout the universe's history^15,16^.

The nature of variations in α and $G$ with respect to space or time is inherently model-dependent, assumed by the following relationship: $\dot{\alpha }/{\alpha }^{2}\sim \dot{G}/G$^17^. Numerous studies have investigated the values of α, often examing the universe's history. Many of these studies have have employed Cosmic Microwave Background (CMB) anisotropy data to discern spatial and temporal variations in α during the early universe^18–20^. High-resolution quasar spectra within absorption systems have provided evidence for smaller variations in the fine-structure constant, α, across redshifts 0.5 < z < 3.5^21^, suggesting that fundamental constants may evolve over cosmic space–time.

The assessment of cosmic space–time variation of these constants is significant. Such variations influence local measurements, including systems like our Sun, the solar system, or the solar neighborhood, and the framework of Big-Bang nucleosynthesis. Lunar Laser Ranging has provided on the time variation of the gravitational constant $\dot{G}/G=(0.2\pm 0.7)\times {10}^{-12}{\text{ yr}}^{-1}$^22^. More robust findings from Big-Bang nucleosynthesis suggest $-0.3\times {10}^{-12}{\text{ yr}}^{-1}\le \dot{G}/G\le 0.4\times {10}^{-12}{\text{ yr}}^{-1}$ ^23,24^. Investigations using the Hubble diagram of Type Ia supernovae have positioned $G$ with a weaker constraint: $\dot{G}/G\sim 1\times {10}^{-11}{\text{ yr}}^{-1}$ at $z\sim 0.5$^25,26^. Recent updates have refined these limits to $-(1.10\pm 1.07)\times {10}^{-12}{\text{ yr}}^{-1}<\dot{G}/G<0$. The most current results indicated $-0.6\times {10}^{-12}{\text{ yr}}^{-1}\le \dot{G}/G<0$^27–33^. Advanced methodologies using astrophysical observations detect space–time variations in fundamental constants, including the fine-structure constant, $\Delta \alpha /\alpha = \left( {0.027 \pm 0.832} \right) \times 10^{ - 6}$ and the proton-to-electron mass ratio, $\Delta \mu /\mu = \left( {0.025 \pm 0.262} \right) \times 10^{ - 7}$. This approach provides stringent constraints and allows a broad evaluation of analytical and systematic errors with high precision^34–36^.

The aim of this study is to utilize a combined wavelength of Ritz in the laboratory and [Fe II] wavelengths from the HE 0515–4414 quasar to investigate potential variations in the gravitational constant $G$ over space and time. Our analysis provides an estimate of the cosmological deviations in the gravitational constant $\dot{G}/G=(0.918 \pm 2.830)\times {10}^{-15}{\text{ yr}}^{-1}$. In any case, this result could significantly improve upon the previously published results^25–33,37,38^.

## Determination of $\dot{{\varvec{G}}}/{\varvec{G}}$ with quasar spectra

The redshifted spectra of quasars offer a valuable tool for investigating spatial and temporal variations in fundamental dimensionless constants, such as the fine-structure constant α and the proton-to-electron mass ratio µ, across cosmic timescales. These constants exhibit sensitivity to change, manifested in resonance states of ions and molecules transitioning to their ground states within these systems. By comparing observed wavelengths with their laboratory values, variations in constants like αcan be directly detected across the universe. Notably, the separation between energy levels, including fine-splitting related to ${\alpha }^{4}$ and the maximum energy level ${\alpha }^{2}$, is proportionally associated with the redshift. This coupling is fundamentally found in the general relativistic effects of redshift $(z)$. It can be quantified by the energy $(E)$ loss of a photon, represented as $z=-\Delta E/E$, where $\Delta E$ is the initial energy of the photon. Consequently, this fractional change in energy corresponds to a fractional change in wavelength observations $-\Delta E/E=\Delta \lambda /\lambda \sim \Delta \alpha /\alpha$^39^. Thus, any variations in α can be directly detected across the expanse of the universe by comparing observed wavelengths with their laboratory values^34–39^.

Based on the context of Grand Unification Theories (GUTs), we explore the assumption that spatial or temporal variations in fundamental physical constants could provide the potential to unify gravitational and electromagnetic forces. Within this framework, transmutable dimensions are used to determine the weak scale, and all related Yukawa couplings exhibit corresponding variations. We assume that these variations are driven by a dilaton-type mechanism, which in turn impacts the fine-structure constant and the Quantum Chromodynamics (QCD) scale :

1 $$ \Delta \Lambda_{QCD} /\Lambda_{QCD} = R\Delta \alpha /\alpha $$

Here, $R$ can be determined by GUTs. Furthermore, we can derive the value of $R$ through a model-independent approach at low energies, driven by the relationship $\alpha \left({M}_{GUT}\right)={\alpha }_{s}({M}_{GUT})$^34–40^. Moreover, grounded in the concept of dimensional transmutation within the weak scale, we determine that significant variations in Yukawa coupling ($h$) lead to corresponding changes in the Higgs vacuum expectation value ($h$).

This, in turn

2 $$\nu ={M}_{Planck}\text{exp}(-\frac{{8\pi }^{2}\text{c}}{{h}^{2}})$$

and

3 $$\frac{\Delta \nu }{\nu }=16{\pi }^{2}c\left(\frac{\Delta h}{h}\right)=S\left(\frac{\Delta h}{h}\right),$$

determines the value of ($\nu$) at the Planck scale based on the GUTs of mass. Consequently, we can derive values for

4 $$\frac{\Delta \nu }{\nu }=S\left(\frac{\Delta h}{h}\right),$$

where $S \equiv d ln \nu /d ln h,c \simeq \hbar \simeq 1$ and $\Delta h/h = (1/2)\Delta \alpha /\alpha$ by extension, the electron mass and the proton-to-electron mass ratio variations as

5 $$\Delta {m}_{e}/ {m}_{e}=1/2(1+S) \Delta \alpha /\alpha $$

6 $$ {\text{and}}\;\Delta m_{p} / m_{p} = \left[ {0.8R + 0.2\left( {1 + S} \right)\left( {\frac{\Delta \alpha }{\alpha }} \right)} \right] $$

As a result, through a perturbative approach, we can obtain variations in the neutron mass ${m}_{n}$ and the average nucleon mass ${m}_{N}$, represented as $\Delta m_{n} /m_{n} = \Delta m_{N} /m_{N} = \Delta m_{p} /m_{p}$^34–40^. Collectively, these investigations allow us to infer changes in both α and $G$ over cosmic space–time, their interrelation and the probability of these variations through an in-depth analysis^41^.

7 $$\frac{\Delta G}{G}=[1.6R+0.4\left(1+S\right)]\frac{\Delta \alpha }{\alpha }$$

In this study, we introduce a set of free phenomenological parameters ($\text{R},\text{ S})$ that are intricately linked to both the Quantum Chromodynamics (QCD) and Electroweak (EW) sectors. These dimensionless coupling exhibit values across various theoretical models and play a crucial role in guiding our analysis. Laboratory determinations of these parameters may exhibit either similar or opposing signs, influencing their application in astrophysical observations. Based on our determined values for $\text{R}=273\pm 86$ and $\text{S}=630\pm 230$, we aim to test the effects of space–time variations in both α and µ with a higher precision. o achieve this, we conduct a comprehensive analysis that couples Ritz wavelengths with observed wavelengths obtained from quasar spectra. We ensure rigorous calibration of wavelengths and quantification of systematic errors during the analytical procedure^42–46^.

We focus our attention on the transition of [Fe II] lines, which are highly suitable candidates for investigating variations in the fundamental constants. These lines are a common observed in quasar spectra and exhibit exceptional sensitivity to variations in essential dimensionless constants, such as the fine-structure constant, the proton-to-electron mass ratio, and the gravitational constant. Their sensitivity often surpasses that of other lines, such as [C I] and [O I]^47^. The advantage of employing [Fe II] lines lies in their consistent line shape, simplifing the definition of parameters used in our analysis and reducing systematic effects. Moreover, the ionization structure or observed substructure of [Fe II] lines is comparatively moderate to other isons, further enhancing their suitability for our anlysis^47^.

**Figure 1:**
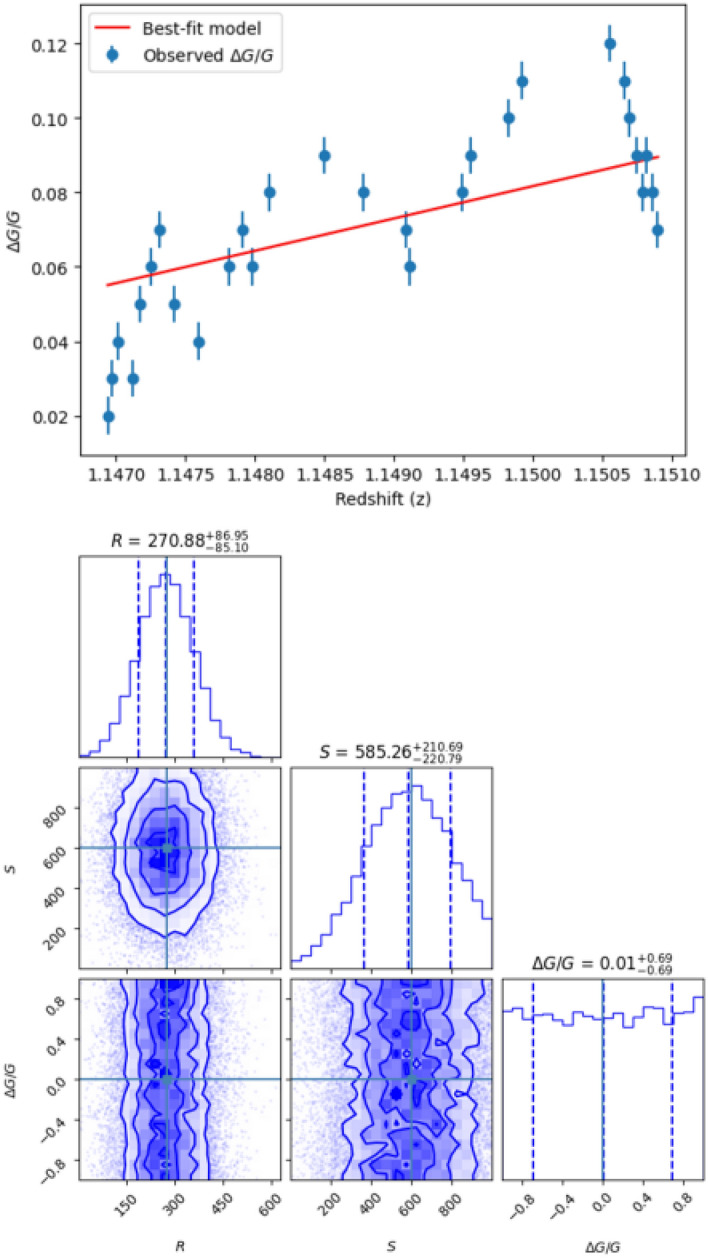
The plot is for multi-parameter fitting of $S$ $R$, and $\Delta \text{G}/\text{G}$ using MCMC sampling. The distributions and covariances of the parameters are shown with 1-sigma (dark blue) and 2-sigma (light blue) confidence intervals. The dashed lines in the histograms represent the median values and the 1-sigma confidence range. The true values used for the observational data are $\text{R}=273\pm 86$ and $\text{S}=630\pm 230$.

Our analytical approach involves combining updated uncertainty error estimations with high-quality [Fe II] spectra^48,49^ to achieve a high level of accuracy in estimating systematic errors. We utilize the combined wavelengths of [Fe II] lines derived from quasar spectra and Ritz wavelengths from laboratory measurements. The energy levels relative to [Fe II] are precisely determined through Fourier transform spectroscopy. In our analysis, we indicated that the Doppler shift is used to determine the velocity scale of [Fe II] line spectra derived from quasar observations. In the context of this choice is that the selected [Fe II] lines exhibit uniform velocities, having the same shape, and are characterized by their narrow profiles. To accurately determine their central velocities and linewidths, we employed Gaussian-fitting techniques. Our approach involved the application of a single-fitting Gaussian for single-velocity components and multiple-fitting Gaussians for cases involving multiple-velocity components. Thus, the components of [Fe II] lines were characterized based on their Doppler shift, column density $N$, absorption redshift ${z}_{abs}$, and linewidth $b$. Progressing step by step, we used the [Fe II] lines to estimate the values $\Delta \alpha /\alpha$. These values are then intergrated with fitting parameters (R, S, $\Delta \alpha /\alpha$) to derive the values of $\dot{\text{G}}/\text{G}$. Our analytical methodology employs a non-linear least-squares fitting approach, combining laboratory measurements with an uncertainty of ${10}^{-6}$ and observed wavelengths with an uncertainty of ${10}^{-7}$. This approach enables precise estimation of the effects of time-variation in the gravitational constant over cosmic timescales. Moreover, we employ a multi-parameter joint analysis approach within a Bayesian framework to comprehensively assess the uncertainties associated with the derived parameters, including $\Delta \alpha /\alpha$, R, S, and $\Delta {\text{G}}/{\text{G}}$. This approach allows us to capture the correlations between different parameters and provides a more complete characterization of the parameter space. We begin by defining the parameters of interest and selecting prior distributions based on previous studies and theoretical considerations. These priors guide the analysis and help constrain the parameter space. Using observed data and a likelihood function constructed from observational uncertainties and model assumptions, we derive posterior distributions for each parameter using Bayes' theorem. Markov Chain Monte Carlo (MCMC) sampling techniques are then employed to explore the parameter space and sample from the posterior distributions. The resulting posterior distributions provide insights into the uncertainties of each parameter and quantify the correlations between different parameters, enriching the interpretation of the results in Fig. 1. Finally, we utilize model comparison techniques within the Bayesian framework to assess the goodness-of-fit of different parameter models and select the most suitable model for describing the data^50–53^.

**Figure 2:**
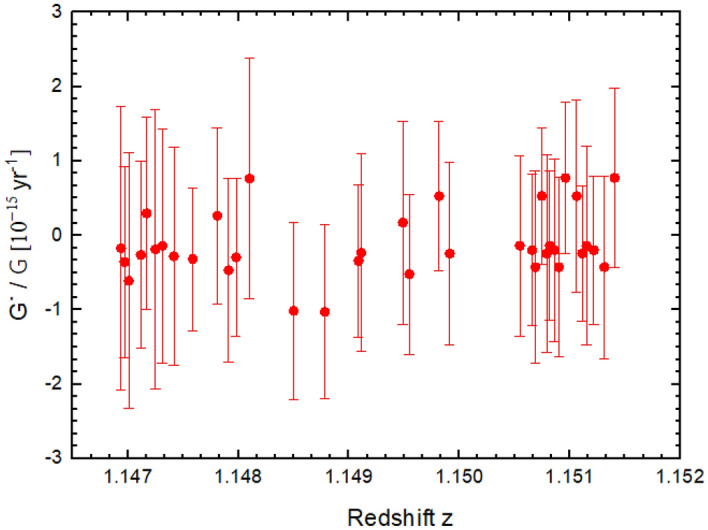
Illustration of time-variation in gravitational constant $\dot{G}/G$, with redshift.

In our analysis, we focuse on changes in $\dot{G}/G$ using both minimal (${\chi }^{2}$) and maximal (${\chi }_{min}^{2}$) values with fitting-approximated reductions (${\chi }^{2}\simeq 1$). We employ $\Delta {\chi }^{2}= {\chi }^{2}-{\chi }_{min}^{2}=1$ to evaluate the variation of $\dot{G}/G$, deriving maximal variations to simplify the estimation of associated errors. Our study yieldes an upper limit on the time variation of the gravitational constant $\dot{G}/G=(0.918 \pm 2.830)\times {10}^{-15}{\text{ yr}}^{-1}$, as detailed in Table 1. We estimate both statistical and systematic errors (${\sigma }_{tot}^{2}= {\sigma }_{\dot{G}/G}^{2}+{\sigma }_{sys}^{2}$), representing the *G* variation with redshift in Fig. 2.

**Table 1:** The determination of $\dot{G}/G$ was based on the combined Ritz wavlelength of [Fe II] in laboratory and QSO HE 0515–4414^*^

${\uplambda }_{\text{Ritz wavelength}}$	${\uplambda }_{\text{Obser}}$	${\text{z}}_{\text{abs}}$	$\dot{G}/G[{10}^{-15}{\text{ yr}}^{-1}]$	${\sigma }_{\dot{G}/G [{10}^{-15}{\text{ yr}}^{-1}]}$
1608.29739	3453.2908 (57)	1.14694	− 0.17294	− 0.42952
1608.45082	3456.6993 (39)	1.14697	− 0.35839	0.28921
1608.53706	3459.2892 (37)	1.14701	− 0.61080	− 0.38614
1609.03712	3459.4431 (11)	1.14712	− 0.26460	− 0.28308
1609.02760	3459.7131 (13)	1.14717	0.29625	0.28950
1609.09965	3460.2938 (17)	1.14725	− 0.18797	0.42348
2344.15461	5032.9469 (24)	1.14731	− 0.14136	0.35475
2344.28128	5037.9119 (23)	1.14742	− 0.28118	− 0.32821
2344.46761	5041.6969 (25)	1.14759	− 0.31963	− 0.21558
2344.46306	5041.9145 (12)	1.14781	0.26209	− 0.26589
2344.60282	5042.3034 (10)	1.14791	− 0.46950	0.27880
2344.79566	5043.1547 (15)	1.14798	− 0.29768	0.23813
2374.07080	5102.9147 (36)	1.14810	0.76227	0.36314
2374.12864	5106.7434 (36)	1.14850	− 1.01580	− 0.26726
2374.12444	5106.9734 (12)	1.14878	− 1.02899	0.26329
2374.20858	5107.3695 (16)	1.14909	− 0.34307	− 0.23065
2374.34557	5108.2272 (21)	1.14911	− 0.23495	0.29878
2383.06018	5115.7159 (17)	1.14949	0.17044	− 0.30683
2383.11729	5120.7677 (21)	1.14955	− 0.52244	0.24181
2383.14402	5124.6089 (22)	1.14982	0.52620	− 0.22549
2587.08205	5553.4531 (29)	1.14992	− 0.24585	− 0.27591
2587.17085	5558.9313 (28)	1.15055	− 0.14076	− 0.27231
2587.36340	5563.1057 (30)	1.15066	− 0.20057	0.22867
2587.64088	5563.3452 (11)	1.15069	− 0.42734	− 0.29076
2587.94910	5563.7764 (11)	1.15075	0.52620	− 0.20508
2588.19112	5564.7129 (16)	1.15079	− 0.24585	0.29971
2600.15976	5582.4812 (19)	1.15082	− 0.14076	− 0.22549
2600.16977	5587.9914 (21)	1.15086	− 0.20057	− 0.27591
2600.37246	5592.1855 (24)	1.15090	− 0.42734	− 0.27231

The result was calculated using the weighted average of all the lines.

Figure 2 provides a detailed representation of the effect of time-variation in the gravitational constant $\dot{G}/G$, utilizing [Fe II] data lines extracted from observed HE 0515–4414 quasar spectra in combination with Ritz wavelengths. Each data point corresponds to a distinct minimal redshift value. Our analysis methodology is based on independent line ratios associated with and the characteristic line shapes of [Fe II]. These line shapes accquire minimal separation, providing a precise estimation of errors, which includes both statistical and systematic errors. The precision of our error estimates is significantly enhanced by evaluating the split-wavelengths for all line pairs of [Fe II]. Thus, we can establish the best value of $\dot{G}/G$ by providing a computational analysis of its sensitivity to time variations concerning ${\chi }^{2}$. In this context, the maximum variation in $\dot{G}/G$ was calculated by ${\chi }^{2}-{\chi }_{min}^{2}=1$, incorporating an error estimation approach as follows: $\Delta {\chi }^{2}= 1$. Subsequently, the smallest value of ${\chi }^{2}$ was selected for each fitting procedure, and this value was consistently applied across all fits, with a standard deviation ${\sigma }_{tot}^{2}= {\sigma }_{\dot{G}/G}^{2}+{\sigma }_{sys}^{2}$ serving as the basis for error estimation in the weighted mean calculation.

## Discussions and conclusions

Astrophysical observations have proven invaluable in investigating of variations in fundamental physical constants. Recent studies have revealed distinct values for the time-dependent impact on the gravitational constant across various timescales^21,55^. Notably, research involving the pulsating white dwarfs G117-B15A and R548 yielded results of $\dot{G}/G\sim -1.8\times {10}^{-10}{\text{ yr}}^{-1}$ and $\dot{G}/G\sim -1.3\times {10}^{-10}{\text{ yr}}^{-1}$^28,55,56^, respectively, utilizing white dwarf asteroseismology $\dot{G}/G=1.3\times {10}^{-10}{\text{ yr}}^{-1}$ as a key analytical method. This constraint could be further refined through the application of white cooling theory, suggesting $\dot{G}/G\le {10}^{-10}-{10}^{-11}{\text{ yr}}^{-1}$^57^. Additionally, studies exploring the galactic cluster NGC6791 have reported findings of $\dot{G}/G\sim -1.8\times {10}^{-12}{\text{ yr}}^{-1}$^58^. Diverse outcomes emerged from investigations involving the pulsar binary system PSR1913 + 16 within the framework of Brans-Dicke theory. Subsequent reevaluations, including data from PSR B1855 + 09, produced results of $\dot{G}/G=(1.0 \pm 2.3)\times {10}^{-11}{\text{ yr}}^{-1}$ and $\dot{G}/G=\left(1.0 \pm 2.3\right)\times {10}^{-11}{\text{ yr}}^{-1}$^59–61^. To date, updated findings estimate this effect at a level of ${10}^{-12}{\text{ yr}}^{-1}$. Certain studies have reported $\left|\dot{G}/G\right| <1.6\times {10}^{-12}{\text{ yr}}^{-1}$ and $\dot{G}/G=(-0.6 \pm 4.2)\times {10}^{-12}{\text{ yr}}^{-1}$ at the 2σ by confidence level, utilizing data from six telescopes with p-mode spectra^62–66^.

In this study, we have presented a potential cosmological variation in the gravitational constant across cosmic timescales. Our analysis has yielded a critical finding $\dot{G}/G=(0.918 \pm 2.830)\times {10}^{-15}{\text{ yr}}^{-1}$, utilizing the combined wavelengths of [Fe II] and Ritz wavelengths. This result represents the most robust limits on the gravitational constant across various theoretical models, such as Pulsar timing, Lunar Laser Ranging, Big Bang nucleosynthesis, and age of globular clusters^62–66^. Notably, this limit has the potential for even better accuracy compared to current studies through the careful selection of candidates, such as CH_3_OH, OH, and CH molecules^67–73^.

Future analyses leveraging high-resolution data from astrophysical observations hold the promise of yielding the most reliable estimates of upper bounds on potential spatial and temporal variations of the gravitational constant. Moreover, forthcoming laboratory experiments and enhanced quality of observational data will further constrain variations in dimensionless physical constants. Regardless, this study serves as a valuable tool for testing parameters within unification scenarios^74–76^.

## Data Availability

The data used to support the findings of the present study are listed in Table 1. All data generated or analysed during this study are included in this published article (10.1051/0004-6361:20054584).
